# *Mycobacterium tuberculosis* Dormancy: How to Fight a Hidden Danger

**DOI:** 10.3390/microorganisms10122334

**Published:** 2022-11-25

**Authors:** Elena G. Salina, Vadim Makarov

**Affiliations:** Bach Institute of Biochemistry, Research Center of Biotechnology of the Russian Academy of Sciences, Moscow 119071, Russia

**Keywords:** *Mycobacterium tuberculosis*, dormancy, latency, “non-culturability”, resuscitation, persistence

## Abstract

Both latent and active TB infections are caused by a heterogeneous population of mycobacteria, which includes actively replicating and dormant bacilli in different proportions. Dormancy substantially affects *M. tuberculosis* drug tolerance and TB clinical management due to a significant decrease in the metabolic activity of bacilli, which leads to the complexity of both the diagnosis and the eradication of bacilli. Most diagnostic approaches to latent infection deal with a subpopulation of active *M. tuberculosis*, underestimating the contribution of dormant bacilli and leading to limited success in the fight against latent TB. Moreover, active TB appears not only as a primary form of infection but can also develop from latent TB, when resuscitation from dormancy is followed by bacterial multiplication, leading to disease progression. To win against latent infection, the identification of the Achilles’ heel of dormant *M. tuberculosis* is urgently needed. Regulatory mechanisms and metabolic adaptation to growth arrest should be studied using *in vitro* and *in vivo* models that adequately imitate latent TB infection in macroorganisms. Understanding the mechanisms underlying *M. tuberculosis* dormancy and resuscitation may provide clues to help control latent infection, reduce disease severity in patients, and prevent pathogen transmission in the population.

## 1. Introduction

The complexity of tuberculosis (TB) infection is largely determined by the population heterogeneity and the wide spectrum of different metabolic stages, including latent TB (LTB) infection [1]. Latency is a clinical term describing an asymptomatic form of TB infection associated with the presence of dormant, non-replicating, drug-tolerant bacterial populations in the infected macroorganism [2,3] that has a number of cytological and physiological features caused by the adaptive response of bacilli to the immune-mediated defense mechanisms of the host [4].

Currently, LTB is diagnosed using the tuberculin skin test (TST) and the interferon gamma release assay (IGRA) [5]. The TST estimates the immune reaction to purified *M. tuberculosis* proteins, the T-cell response in particular, and the IGRA measures the amount of IFN-γ released from sensitized lymphocytes in response to *M. tuberculosis*-specific antigens in the absence of clinical manifestations or radiological evidence of TB infection. Unfortunately, neither the TST nor IGRA can accurately differentiate between LTB and active TB, distinguish reactivation from reinfection, or distinguish the various stages within the spectrum of *M. tuberculosis* infection [5]. Moreover, both the TST and IGRA have reduced sensitivity in immunocompromised patients and have low predictive value for progression to active TB [6]. 

LTB infection is believed to be successfully eliminated after 9 months of isoniazid or 4 months of rifampicin monotherapy [7,8]. However, a clinical trial discovered that a combination of rifapentine and isoniazid taken weekly for only 3 months was as effective as the standard isoniazid regimen for 9 months taken daily [9]. Recently, it was shown that shortening the duration of treatment might significantly benefit the compliance of latently infected individuals. In particular, a 4-month regimen of rifampicin was recently proposed as the best choice for treating LTBI [10]. These therapeutic recommendations may cause some doubts, as isoniazid is expected to be weakly effective at curing LTB infection a priori as it targets the processes of mycobacteria cell wall biosynthesis, which is obviously inactive in dormant bacilli [11]. The role of isoniazid in such a therapeutic regimen could be to eliminate dividing *M. tuberculosis* bacilli, which appear as a result of the resuscitation of dormant cells during the reactivation of a latent infection [12]. A significant tolerance of dormant cells to rifampicin was demonstrated in the model of paucibacillary infection in mice (or Cornell model) [13], calling into question the effectiveness of rifampicin in the treatment of latent TB. Moreover, the phenotypic resistance of dormant *M. tuberculosis* to the sterilizing action of rifampicin was proposed as a characteristic feature of latent TB [14].

**Figure 1:**
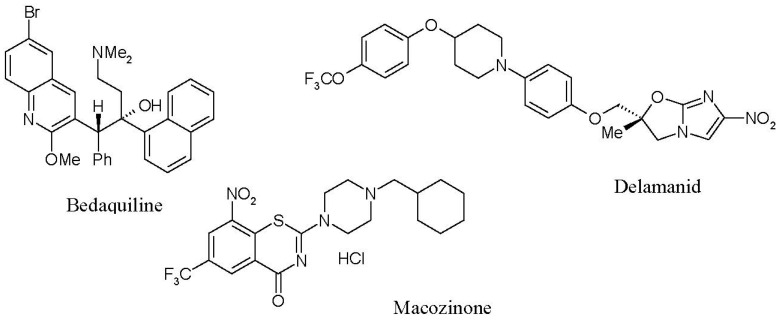
Chemical structures of novel antitubercular compounds.

Recent successes in the development of new anti-tuberculosis drugs with original targets include (i) bedaquiline (Sirturo^®^, Janssen Therapeutics, Titusville, NJ, USA), a specific inhibitor of mycobacterial ATP synthase [15]; (ii) delamanid (Deltiba^®^, Otsuka Pharmaceuticals, Tokyo, Japan), an inhibitor of the biosynthesis of methoxymycolic and ketomycolic acids [16] that is already approved for use in a number of countries; and (iii) macozinone (PBTZ169), which affects the biosynthesis of the mycobacterial cell wall [17,18] by inhibiting the original target of decaprenylphosphoryl-β-D-ribose-2′-epimerase (DprE1), which is currently in the second phase of clinical trials (Figure 1). However, none of these solve the problem of latent TB therapy, since these drugs, which are highly active against replicating *M. tuberculosis*, are ineffective against latent infection [19,20]. Thus, a lack of specific and highly effective anti-latent TB drugs is one of the main reasons that the modern therapy of LTB infection continues to apply conventional antibiotics [20].

Indeed, latent TB infection presents a constant risk of disease reactivation, which is 10% over a lifetime in the general population and 10% per year in the case of those with a compromised immune system due to HIV infection, undernutrition, smoking, diabetes, etc. [21,22,23,24]. Although from a clinical point of view the reactivated TB form cannot be differentiated from the primary disease, epidemiological studies show that most cases of active TB in countries with low levels of TB prevalence are the result of the reactivation of latent infection.

Thus, the early prevention of LTB reactivation, especially for people with increased risk of the transition from LTB to active disease, e.g., those with HIV/AIDS and those receiving immunosuppressive therapy, steroid treatment, or tumor necrosis factor (TNF-α), as well as persons with chronic systemic diseases such as terminal stage renal disease, rheumatic disorders, or diabetes mellitus, is one of the top priorities in science today [25]. To find new drugs against LTB, a more thorough approach to the modeling of latent infection and a deeper study of the forms and mechanisms of persistence of *M. tuberculosis*, as well as the reactivation of latent infection, are needed.

## 2. Challenges in Modeling LTB Infection

Several *in vivo* models of *M. tuberculosis* latency and reactivation have been developed to date, including in mice [26,27,28], guinea pigs [29], and rabbits [30,31]; however, they do not fully reproduce the disease pathology and immune control observed in humans. The composition of murine TB granulomas is quite similar to human granulomas; however, mouse TB lesions lack tissue necrosis, which is the pathological hallmark of human TB granulomas [32]. Granulomas in guinea pigs and rabbits more adequately represent the human granuloma; while TB granulomas in standard mouse models are not hypoxic, guinea pigs and rabbits are characterized by tissue hypoxia [32]. TB models in non-human primates most closely illustrate human TB [33,34,35]; however, they are expensive and time-consuming. 

*In vitro* modeling is a much more cost-effective strategy, which enables the investigation of the *M. tuberculosis* resuscitation process in detail and may identify principal molecular players, providing necessary clues for further *in vivo* experiments [35]. However, despite extensive attempts to model *M. tuberculosis* dormancy *in vitro*, including the well-known Wayne model of the non-replicating state under progressive hypoxia [36,37], the dormant bacilli obtained in the majority of *in vitro* models were fully culturable, whereas bacteria isolated from *in vivo* models of latent TB are “non-culturable” [13,38]. “Non-culturability” is a specific term for cells that are temporarily unable to grow on standard solid media and become culturable only after a special procedure of resuscitation [39]. Therefore, to imitate latent TB in humans and animals more adequately, *in vitro* models of *M. tuberculosis* dormancy should reproduce the phenomenon of “non-culturability”. Recently, a new *in vitro* model of *M. tuberculosis* dormancy and “non-culturability” in K^+^-limiting conditions was proposed, in which the bacteria acquired high tolerance to rifampicin and isoniazid. Remarkably, dormant bacilli in this *in vitro* model developed a temporary “zero-CFU” phenotype and were characterized by significant (up to 1 × 10^7^ cells/mL) recovery potential [40,41] after the abolition of potassium deficiency.

Despite the disadvantages of individual models of LTB infection, modeling the infectious process both *in vivo* and *in vitro* has played an important role in the development of TB drugs and vaccines and has given us a deeper understanding of TB immunopathogenesis. Here, we give a short overview of the most popular models of LTB developed to date, their advantages, and their disadvantages. For more information on LTB modeling, we recommend some recent comprehensive reviews [3,34,42,43,44,45].

### 2.1. Murine Models

The modeling of LTB in mice has been deeply studied in comparison to other *in vivo* models due to the advantages of predictability, simple handling, low cost, availability of immunological tools, and availability of inbred, outbred, and transgenic strains [44]. Murine models allow for the study of different mechanisms of the host’s immune protection, which is also important for understanding LTB infection in humans, even though some of the detailed pathways in murine models and humans may differ [42,43].

Model of chronic TB. Unlike LTB in humans, a classic murine model of chronic *M. tuberculosis* infection is characterized by a high bacterial load with progressive pulmonary disease and the premature death of the animals [46]. Traditionally, the stable level of bacilli in the mice’s lungs at the chronic stage of infection was perceived as a static balance, based on the fact that bacteria in this state have reduced metabolic activity and replicate very slowly [47]. This experimental approach involves intravenous injections of *M. tuberculosis* at different doses. For approximately 2 weeks after infection, the bacilli actively divide in the lungs. After this, the number of bacteria remains stable for at least 80 days. This plateau corresponds to 4 × 10^4^ ÷ 4 × 10^6^ CFU, depending on the number of bacteria used for the infection. It is assumed that these bacteria are in a specific, close-to-dormant state, and their transition to an inactive state is related to the host’s immune response to the infection [48,49]. However, during chronic TB infection, bacteria are able to proliferate further. For example, Orme et al. [48,50,51], infected 3-month-old mice with a low dose of bacteria (CFU ≤ 10) and monitored the development of the infection over the animals’ whole lifecycle. After the initial proliferation in the lungs, followed by the transition to a non-dividing state, the bacteria stayed inactive until the age of 18 months. After that, the bacilli started to proliferate rapidly, which led to the death of the animals. The reactivation of the bacteria in 18-month-old mice corresponded with age-related changes in the immune response [48,52]. Nevertheless, in chronically infected mice, *M. tuberculosis* demonstrates substantial antibiotic resistance to isoniazid in comparison to the acute phase of infection (0—14 days after infection) [53].

The Cornell model of paucibacillary infection. In 1956, McCune and Tompsett from Cornell University developed a model of paucibacillary infection where mice infected with *M. tuberculosis* received isoniazid in combination with pyrazinamide. Treatment started on the day of intravenous infection, lasted for 30 days, and resulted in the clearance of *M. tuberculosis* from the lungs and spleen. However, 19 days after the end of the 30-day treatment course, viable bacteria re-appeared in the tissues of 30% of mice [26,54]. 

In 1995, de Wit and co-workers [55] used large *M. tuberculosis* inoculates and low doses of pyrazinamide (lower than in the Cornell model) to show that, after 12 weeks of antibacterial therapy, the colony-forming unit (CFU) numbers decreased from 10^7^ per organ to 0. However, qPCR data estimated about 5.5 log10 bacilli equivalents in the spleens and lungs. It was suggested that a major proportion of the bacterial population may be represented by “non-culturable” bacilli [55]. However, due to the absence of experiments for resuscitation, there may be some difficulties with the interpretation of the data obtained in the Cornell model, as the increase in CFU numbers [26,54] might also reflect the proliferation of a very low amount of viable cells that might persist after treatment with antibiotics [56]. Although the Cornell model of paucibacillary infection has been used to study the immunological mechanisms of LTB infections [27], it might be more correctly described as a model of *M. tuberculosis* persistence because the low infectious load is reached by antibiotic treatment and not by the host’s immune response.

Model of immune containment of infection. In recent years, a mouse model of the containment of *M. tuberculosis* infection by host immunity, which could overcome the problem of high bacterial load in the lungs leading to the premature death of infected mice, has been developed. In the most successful version of this model, mice were injected with a recombinant BCG strain overexpressing the 30 kDa major secretory protein [57], and after 6 weeks they were infected with a virulent *M. tuberculosis* H37Rv [58], which led to a stable level of bacilli in the lungs at 10^4^. Later, this model was transferred to C3Heb/FeJ mice, leading to the development of necrotic granulomas in the lung, meaning that the infection may reactivate under anti-TNF-α antibody treatment [59]. This model differs from LTB infection in humans because mice need a preliminary vaccination for the immune regulation of asymptomatic paucibacillary infection.

Artificial granuloma model. A relatively novel murine model of an artificial hypoxic granuloma involves the encapsulation of *M. tuberculosis* in semidiffusible hollow fibers that are implanted into mice subcutaneously [60]. After a while, a granuloma containing mycobacteria with decreased metabolic activity develops around such a capsule. These mycobacteria show an antimicrobial susceptibility pattern similar to persistent bacilli obtained under chemotherapy [60]. Although these artificial granulomas are not localized in the lungs, this approach has several advantages in comparison to other *in vivo* models. First of all, it is simpler and more convenient for experimental manipulation when studying the mechanisms of bacterial adaptation to the host’s immune system and the characteristics of persisting mycobacteria. In particular, these mycobacteria demonstrated the induction of dosR (Rv3133c) and 20 other members of the DosR regulon involved in the regulation of dormancy survival. Interestingly, the dormancy phenotype of extracellular *M. tuberculosis* within host granulomas appears to be interferon-gamma dependent. Interestingly, although in this model the granulomatous tissue is hypoxic, metronidazole, which was effective against dormant cells in the Wayne *in vitro* model of progressive hypoxia [61], did not affect these non-replicating cells. The extracellular location of *M. tuberculosis* in hypoxic granulomas is a unique characteristic of this model that matches the features of *M. tuberculosis* populations during LTB in humans [60].

The model of infection in C3HeB/FeJ mice. Judging from the histopathological criteria, the standard murine model produces a granuloma that substantially differs from that in humans. Even so, the cellular content of granulomas in mice and humans are similar, except for the absence of giant cells with several nuclei in mice and the fact that the lesions in mice are poorly organized and represent a mixture of activated and epithelioid macrophages and lymphocytic clusters [62]. In addition, the TB lesions in mice are not characterized by tissue necrosis and hypoxia, which is a characteristic trait of TB granulomas in humans. Moreover, it is believed that LTB infection in humans represents a wide spectrum of various physiological states of bacilli, from replicating to dormant “non-culturable” *M. tuberculosis*. Several attempts to develop a murine model that illustrates *M. tuberculosis* infection in humans [63] more adequately has led to the development of C3HeB/FeJ mice, also known as the Kramnik model [64]. This mouse model is believed to mimic human pulmonary TB lesions most adequately [64]. By positron emission tomography in live infected animals, postmortem immunohistochemistry with pimonidazole, and an analysis of bacterial gene expression, it was demonstrated that the TB lesions in C3HeB/FeJ mice were hypoxic at later stages of infection [65]. This is the main difference in terms of tuberculosis infection in C3HeB/FeJ mice in comparison with the BALB/c line, which are more common in experiments on tuberculosis. In contrast to BALB/c mice, the combination of moxifloxacin and pyrazinamide did not have a bactericidal effect in C3HeB/FeJ mice. Although adding PA-824 slightly increased the efficacy of treatment, this experimental combination of PA-824 and pyrazinamide was less effective in C3HeB/FeJ mice than a standard regimen [66,67]. Thus, the authors demonstrated that conventional anti-TB drugs were significantly less active in C3HeB/FeJ mice than in BALB/c mice. Indeed, the model of tuberculosis in C3HeB/FeJ mice demonstrates key features of LTB in humans and may be recommended as a relevant model for preclinical testing of new anti-TB compounds and vaccines [68].

### 2.2. Guinea Pig and Rabbit Models

In contrast to mice, granulomas in guinea pigs and rabbits are more similar to granulomas in humans in terms of cellular composition, granuloma morphology, tissue hypoxia, and the presence of caseous necrosis [69,70,71]. In addition, the guinea pig model of TB distinguishes between primary granulomas and secondary lesions, which are thought to be the result of hematogenous spread [72]. However, just as in mice, *M. tuberculosis*-infected guinea pigs develop disease with a high bacterial load and are unable to resist infection, suggesting that LTB models in these animals may be difficult to establish [73] and that studying the mechanisms of the immune regulation of TB in these animals is not reliable [74]. 

The infection of rabbits with *Mycobacterium bovis* leads to the development of a disease with a high bacterial load, accompanied by the formation of caseous granulomas, the liquifying of lesions, and the formation of cavities. On the other hand, rabbits are resistant to *M. tuberculosis* infection, and the paucibacillary population in the lungs of animals develops even after 10 weeks of infection and may be reactivated by immunomodulators [75]. 

Since preliminary sensibilization may change the pathology of the disease in rabbits, different *M. tuberculosis* strains may cause a wide spectrum of diseases [76]. Recently, Subbian et al. described a highly promising model of latent TB on New Zealand rabbits, which demonstrated spontaneous and complete clearance of bacilli from the lungs, as well as symptomatic resolution, after 12 weeks of infection with *M. tuberculosis* CDC1551 [77]. Remarkably, these animals are more likely to develop latent TB rather than tissue sterilization, and the administration of systemic corticosteroids may cause TB reactivation [77]. 

### 2.3. Non-Human Primate Models

The model that most closely reproduces the clinical, histological, and microbiological characteristics of LTB infection in humans was developed in non-human primates [78,79]. In contrast to the murine granulomas and similarly to the granulomas in guinea pigs and rabbits, the necrotic granulomas in non-human primates are hypoxic [69]. Although primates infected with high doses of *M. tuberculosis* bacilli (10^4^–10^5^) developed acute, rapidly progressing, and highly lethal pneumonia, infection with a low dose *of M. tuberculosis* (approximately 25 bacilli) via bronchoscopy demonstrated a positive skin tuberculin test in the absence of any clinical symptoms for about 40% of primates at least 6 months post-infection [80].

In contrast to the primates with active disease demonstrating infiltrates or cavities and lesions with a high bacterial load, animals with a latent infection did not demonstrate abnormal chest X-ray findings. Latently infected primates produced small granulomas in the lungs and hilar lymph nodes that contained a small number of bacilli, which were characterized by central caseation, calcification, and peripheric fibrosis. The accessibility of the immunological approach could help significantly with the study of LTB infection and the phenomenon of reactivation in non-human primates; however, the use of this model is limited due to the high cost of the animals and ethical restrictions.

### 2.4. Zebrafish Models

A recent but popular model of mycobacterial infection is a zebrafish model [81,82]. Due to the histological and pathological similarities between *Mycobacterium marinum* infection in zebrafish and human *M. tuberculosis* infection, it is believed that this model can be used to study drugs used against actively growing mycobacteria and latent infection. Its advantages over traditional mammalian *in vivo* models are that it is inexpensive and easy to operate. Thus, a zebrafish model may be useful for evaluating new vaccines against human TB, high-throughput screening for small molecules with anti-TB activity, and the repurposing of previously known drugs for TB [81,83,84]. Despite some evident limitations, *in vivo* models substantially benefit our understanding of the phenomenon of LTB in humans and help elucidate the complex pathways that are involved in host–pathogen interaction, as well as aiding drug discovery and vaccine development [43]. Establishing advanced animal models helps to further advance TB therapy in a well-defined, reproducible, and cost-efficient way.

### 2.5. Models of LTB In Vitro

Reproducing the full spectrum of host–pathogen interactions involved in LTB infection by *in vitro* models is accompanied by obvious limitations, but they are much less expensive and time consuming. Moreover, in comparison to *in vivo* models, *in vitro* models allow us to obtain dormant cells in preparative amounts to study the phenomenon of dormancy in more detail [24]. A wide spectrum of stress factors such as oxygen deprivation, lack of nutrients, acidic surrounding, long-term cultivation in the stationary phase, etc. [14,36,37,40,85,86,87,88,89,90] is being used to induce the dormant *M. tuberculosis* phenotype *in vitro*. However, many *in vitro* dormancy models produce culturable bacteria, while, as was already mentioned, bacteria isolated from the lungs of latently infected mammalians are “non-culturable” [13,38] and require a special procedure of resuscitation to re-start their replication [13,38]. This observation widens the gap between *in vitro* modeling and LTBI infection in humans.

The model of progressive hypoxia. Currently, a model of *M. tuberculosis* dormancy under progressive hypoxia, or the Wayne model, remains the most studied *in vitro* model [37,91]. To obtain dormant bacteria, *M. tuberculosis* bacilli are incubated in tightly closed tubes, adapting to gradual oxygen depletion. When the concentration of the dissolved oxygen decreases to less than 1%, the bacteria reach non-replicative state I (NRP-I), which is characterized by the thickening of cell walls and the cessation of replication and transcription. When oxygen saturation continues to decrease and reaches 0.06%, the bacilli transition to non-replicative state II (NRP-II), which is accompanied by tolerance to isoniazid but not to rifampicin. The key regulator of *M. tuberculosis* dormancy under hypoxic conditions is the two-component regulator DosR (DevR, Rv3133c) [92,93,94], which induces a group of 49 genes called Dos-regulon. The genes of Dos-regulon are also induced not only in response to hypoxia but also in the presence of nitric oxide in *M. tuberculosis* static culture, as well as during the infection of mouse macrophages and in a guinea pig *in vivo* model [95,96,97]. Interestingly, progressive hypoxia makes *M. tuberculosis* sensitive to metronidazole [98] and its derivatives, and metronidazole is also effective in infected rabbits and non-human primates, in which it forms a hypoxic granuloma [99]. A recent study adapted the Wayne model of progressive hypoxia to a high-throughput screening of molecules against latent tuberculosis [100] and provided new perspectives on TB drug development. Notably, dormant cells in the Wayne model of progressive hypoxia remained culturable and started to grow immediately after reintroducing oxygen without a special resuscitation procedure.

The model of enduring hypoxia. Rustad et al. studied the adaptive response of *M. tuberculosis* to prolonged hypoxia. They suggested that the initial hypoxic response of *M. tuberculosis* controlled by the two-component regulator DosR is an intermediate step of the adaptation of mycobacteria to hypoxic stress [101]. The authors believed that a large cohort of genes may be essential for entry into hypoxic dormancy and its maintenance. A microarray analysis of oxygen-starved cultures revealed that the induction of DosR regulon is a transient adaptation step to hypoxic conditions. The enduring hypoxic response (EHR), which involves the significant activation of 230 genes, is followed a DosR-mediated initial hypoxic response. In particular, EHR genes include many transcriptional regulators that could control the program of *M. tuberculosis* viability in the dormant state. The authors suggest re-evaluating the role of the DosR and the initial hypoxic response in the physiology of *M. tuberculosis* [101].

Nutrient deficiency model. A lack of nutrients, which mimics the conditions of a necrotic granuloma, is known to cause bacterial growth cessation in *M. tuberculosis* and a decrease in metabolic activity [102]. Transcriptomic and proteomic studies of *M. tuberculosis* under nutrient deficiency found the repression of energy metabolism, lipid biosynthesis, and cell division and the induction of a stringent response [86]. These starved mycobacteria had decreased sensitivity to rifampicin and isoniazid [103]. However, similarly to hypoxic dormancy, *M. tuberculosis* under nutrient deficiency did not become “non-culturable” and did not require a resuscitation procedure for the restoration of culturability.

A model of multiple stresses. Deb et al. developed a model of dormancy under multiple stress conditions, such as lack of oxygen (5%), increased concentration of CO_2_ (10%), nutrient deficiency (10% Dubos medium), and acidic pH (5.0) [14]. Under these conditions, the growth of the *M. tuberculosis* stopped, the bacteria became rich in lipids but were no longer acid-resistant, and they developed a phenotypic resistance to isoniazid (and to a lesser extent to rifampicin). The analysis of *M. tuberculosis* gene expression in such conditions shows the activation of genes related to stress response accompanied by the repression of biosynthetic metabolic pathways, as well as the processes of transcription and translation.

An ss18b dormancy model. The diversity of chemical libraries containing thousands of compounds means that it is necessary to develop new LTB models that may be adapted for high-throughput screening [104]. Although *M. tuberculosis* bacteria in such screening models cannot meet all the requirements of true dormancy a priori, they may be useful in the initial screening stage of molecules that are active against LTB. An ss (streptomycin-starved) 18b model [105], based on streptomycin-dependent strain 18b that was isolated from the sputum of a patient with tuberculosis resistant to streptomycin therapy [106], is an example of a screening model. The 18b strain was unable to grow *in vitro* in the absence of streptomycin because of the insertion of a cytosine residue in the 16S ribosomal RNA (rRNA) gene, which is responsible for its resistance to streptomycin [107]. Importantly, the 18b strain was unable to divide in the absence of streptomycin and did not lose its viability for several weeks. The ability of the bacilli to divide and grow returned after adding streptomycin to the growth medium. The 18b strain was also used for the development of *in vivo* dormancy models in mice and guinea pigs [108,109]. Interestingly, the non-growing *M. tuberculosis* bacteria in the ss18b *in vitro* model were tolerant to isoniazid and macozinone but not to rifampicin, moxifloxacin, or bedaquiline. 

A model of gradual external acidification. The gradual acidification of the medium during bacterial growth (рН 8.5 → 6.0) was found to lead to the formation of dormant ovoid cells with a changed morphology, a thick cell wall, low metabolic activity, and increased resistance to antibiotics and heating [89]. The ovoid cells lost the ability to form colonies on solid media and became “non-culturable”, making them more similar to LTB infection *in vivo*. In the early stages of acidification, ovoid cells were able to self-resuscitate in a liquid medium; however, after prolonged incubation under low pH, they required a supernatant from an actively growing *M. tuberculosis* culture or an exogenous recombinant Rpf protein for successful resuscitation [89].

A model of “non-culturability” under potassium deficiency. An *in vitro* model of *M. tuberculosis* dormancy and “non-culturability” was developed under potassium-deficient conditions [40,90]. This “zero-CFU population” of dormant *M. tuberculosis* bacilli requires a special procedure of resuscitation in a liquid medium [40]. Dormant “non-culturable” cells were morphologically distinct [90] and considerably tolerant to the first-line antibiotics rifampicin and isoniazid [40]. Transcriptomic and proteomic profiling through adaptation to this dormant “non-culturable” state revealed a switch of bacilli to anaerobic respiration without oxygen limitation [41,90]. The high concordance of the transcriptomic signature in a model of “non-culturability” with transcriptomic signatures of *M. tuberculosis in vivo* models suggested that “non-culturable” mycobacterial phenotypes probably exist during TB disease and may represent unrecognized populations in mammalians [90]. The resuscitation of “non-culturable” bacilli after the re-introduction of potassium was characterized by an immediate transcriptional burst; however, the restoration of culturability did not occur until 7 days after resuscitation [110]. Due to its marked tolerance to both rifampicin and isoniazid, the “non-culturability” model was proposed as a reliable tool for screening drug candidates for curing latent *M. tuberculosis* infection [40].

Evidently, the development of *M. tuberculosis in vitro* dormancy is a rather challenging process. The approaches used for the modeling of *M. tuberculosis* dormancy are constantly evolving from simple approaches involving nutrient deprivation to models that explore multiple stress factors [3]. Table 1 summarizes the range of TB dormancy models *in vitro* developed to date. Despite the fact that the results obtained with the *in vitro* dormancy models cannot be directly transferred to the processes in an *M. tuberculosis*-infected human, *in vitro* models remain one of the main sources for gathering information about the phenomenon of *M. tuberculosis* dormancy. 

## 3. Regulatory Mechanisms of and Metabolic Adaptation to Growth Arrest

Evidently, the ability of *M. tuberculosis* to persist in the human body for a long time is one of the main barriers to the successful eradication of LTB infection. One of the top priorities for modern infection biology is revealing the mechanisms of the maintenance of the latent form of TB, which will help to provide information that can help us to control and combat latent infection.

### 3.1. A Two-Component Regulatory System DosR–DosS

A two-component system, DosR–DosS (also known as DevR–DevS), has been identified as a principal regulator of *M. tuberculosis* hypoxia response [92,111]. The phosphorylation of DosR by any of the two histidine kinase sensors, DosS or DosT, leads to the induction of a cohort of genes named Dos-regulon. DosR and Dos-regulon seem to play a central role in *M. tuberculosis* survival under progressive hypoxia, switching from aerobic metabolism to anaerobic metabolism to sustain energetic resources and redox balance [112]. Recently, Trauner et al. showed that the DosR-regulated protein RafH helps mycobacteria to survive during hypoxia by stabilizing the ribosomes in their associated form [113]. Interestingly, an analysis of the expression of *M. tuberculosis* proteins demonstrated that in comparison to aerobic growth, the changes in expression of ribosomal proteins under hypoxia was quite modest; however, the expression of the components of the electron transport chain and energetic metabolism were affected significantly. Significant changes were also found in the regulation of the biosynthesis of alanine/glutamate and the metabolism of trehalose, accompanied by the activation of lipid metabolism [114].

Interestingly, DosR is not an essential gene for *M. tuberculosis*, as *dosR* knockout causes only a very insignificant decrease in viability under hypoxia [101,115]. A recent hypothesis published by Orme suggests that the activation of DosR is a general strategy of the adaptation of *M. tuberculosis* to unwelcoming surroundings, particularly in necrotic tissue under free active radicals generated by the host’s body [116]. An increased level of the transcription of Dos-regulon has also been found in a dormant aerobic culture of *M. tuberculosis* under potassium deficiency [41,90] and in a model of artificial granuloma in mice [60], which also supports the idea of the activation of Dos-regulon under a spectrum of stress conditions rather than under hypoxia.

### 3.2. Bioenergetics and Lipid Metabolism

Fatty acids are known to be the main carbon source for *M. tuberculosis* during infection and *in vivo* survival [117,118]. The activation of the beta-oxidation of fatty acids, as well as the glyoxylate cycle, was found in bacilli during the infection of macrophages [119] and mice [120], proving the key role of lipids as a source of carbon and energy in the pathogenesis of TB. There is some other experimental evidence that suggests that the accumulation of lipids determines the successful survival of *M. tuberculosis* in the host organism [121]. In addition, the development of the phenotypic resistance of persistent mycobacteria to antibiotics can also be mediated by lipid accumulation. For example, a mutant lacking *tgs1*, which encodes triglyceride synthase, demonstrated a reduced ability to accumulate triglycerides and was characterized by a reduced resistance to antibiotics, and the complementation of this gene restored the resistance of *M. tuberculosis* to antibiotics (Deb et al., 2009).

A recent study on the host’s lipids’ influence on *M. tuberculosis* resistance to antibiotics [122] discovered the possible involvement of the host’s lipids in the formation of antibiotic-resistant dormant mycobacteria. The sensitivity of *M. tuberculosis* to two combinations of antibiotics, rifampicin, moxifloxacin, amikacin, and metronidazole (RIF–MXF–AMK–MTZ) and rifampicin, moxifloxacin, amikacin, and pretomanid (RIF–MXF–AM–PRE), was studied. It was found that both combinations of antimicrobials demonstrated efficacy in *in vitro* cultures when dextrose was used as a carbon source. However, the authors revealed that neither of the two drug combinations revealed a bactericidal effect in media containing cholesterol and lipids. The authors suggested that lipids make *M. tuberculosis* tolerant to antibiotics and that this tolerance is more pronounced in dormancy. 

In a recent publication [123], the authors compared the transcriptional response to hypoxia in *M. tuberculosis* cultures grown on (i) a mixture of long chain fatty acids; or (ii) dextrose as the main source of carbon. Using RNA-seq, the investigators identified differently expressed genes in the phases of early and late hypoxia in the Wayne *in vitro* model and compared these with those identified in the exponential growth phase. The number of upregulated genes in *M. tuberculosis* grown on fatty acids was relatively low in comparison to the cultures grown on dextrose in both the early and late hypoxia phases. The low level of induction of proteins of the stress response in a fatty-acid-rich medium allowed the authors to propose the hypothesis that the lipid environment may decrease the stress that *M. tuberculosis* bacteria suffer under hypoxia [123]. Thus, the presence of lipids in the medium may elicit an adaptive metabolic response from *M. tuberculosis* to diverse types of stress, including hypoxia, and cause the long-term persistence of the pathogen during latent infection. Moreover, mycobacteria are able to synthetize endogenous triacylglycerol using the fatty acids of the host’s tissues [124]. Genes coding for the beta-oxidation of fatty acids by *M. tuberculosis* were upregulated, and the genes of the glyoxylate cycle were found in infected macrophages [119] and mice [120].

### 3.3. The Glyoxylate Cycle

The glyoxylate cycle, which allows for the synthesis of carbohydrates from simple precursors, is induced when fatty acids are the main source of carbon and energy. Interestingly, the activation of the glyoxylate cycle and the utilization of fatty acids as a source of carbon has also been observed in the Wayne dormancy model [96,125,126,127]. The key enzymes of the glyoxylate cycle are isocitrate lyase and malate synthase, which have been found to be essential for successful growth and persistence in macrophages and during acute and chronic mouse infections [117,128]. Moreover, the glyoxylate shunt plays a key role in the survival of the cells in an environment with limited nutrients [129]. Being absent in humans and higher animals, the glyoxylate cycle is an extremely attractive drug target in *M. tuberculosis*.

### 3.4. Stringent Response

Stringent response is one of the general evolutionarily conserved mechanisms allowing bacterial survival in unwelcoming conditions which is mediated by tetra- and pentaphosphate guanosine (p)ppGpp [130,131,132] and affects replication, transcription, and translation. Along with regulating virulence, drug resistance, and biofilm formation, stringent response plays an important role in the establishing chronic *M. tuberculosis* infection [131,133] and in the formation of dormant persister cells [134]. The hallmark of stringent response is the downregulation of rRNA and ribosomal protein synthesis with the concomitant upregulation of amino acid biosynthetic operons to supply the necessary amino acids for survival. The principal mediator of stringent response is the Rel protein; however, CarD-based regulation and inorganic polyphosphate (polyP)-based regulation also affect this signaling pathway [131]. The absence of Rel results in a survival disadvantage to *M. tuberculosis* during stress conditions [131].

### 3.5. Global Transcriptional Repression 

An RNA-seq-based analysis of a dormant “zero-CFU population” revealed at least a 30-fold decrease in the total mRNA level, indicating global transcriptional repression of the protein-coding genes [41]. However, an analysis of “non-culturable” bacilli identified a cohort of mRNA molecules that code for biosynthetic enzymes and proteins involved in the adaptation and repair processes, detoxification, and controlling transcription initiation. The most prominent feature of a transcriptome from *M. tuberculosis* with a “zero-CFU” phenotype was the downregulation of the genes encoding ribosomal proteins. A similar transcriptional signature has previously been observed during starvation [86] in the Wayne model of progressive hypoxia [96], and in persistent mycobacteria after antibiotic treatment [135]. In dormant “non-culturable” bacilli, the decrease in transcripts encoding ribosomal proteins occurred only after the transition to the “zero-CFU” state and not as an early response to K^+^-deficiency, which may represent a specific feature of *M. tuberculosis* adaptation to prolonged dormancy, when only the cells not expressing ribosomal proteins can survive. Remarkably, the entry of *M. tuberculosis* into dormancy was accompanied by the cleavage of the 23S ribosomal RNA between residues G592 and A593. The fragmentation of the 23S rRNA occurred during the initial phase of dormancy and became more pronounced in the late phase of dormancy [41]. During hypoxia, mycobacteria stabilize their ribosomes by keeping the 30S and 50S ribosomal subunits in their associated forms; the inability to stabilize ribosomes results in their degradation and the loss of cell viability [113]. The remarkable transcriptome stability of long-persisting dormant mycobacteria found in this study suggests the existence of effective adaptation mechanisms underlying the readiness of “non-culturable” mycobacteria for resuscitation. The mRNAs in dormant cells may represent a pool of stable transcripts that are rapidly translated upon resuscitation from dormancy. The cleavage of the 23S rRNA at a specific point and the abundance of several small ncRNAs in NC *M. tuberculosis* may indicate their significance for the maintenance of dormancy and suggest a molecular basis for LTB infection.

## 4. Resuscitation from Dormancy: Factors and Triggers

Despite significant recent effort, little is still known about the exact mechanisms of the transition of dormant “non-culturable” *M. tuberculosis* bacilli to the replicating state or about latent TB reactivation. There are at least two reasons for a lack of such information. Firstly, the *in vivo* models of LTB reactivation (particularly the Cornell mouse model, which is well-studied) are mainly focused on the host immune response rather than on the mechanisms of bacterial resuscitation [32,136]. Secondly, the vast majority of *in vitro* studies on the reactivation of dormant bacilli are based on re-aeration in the Wayne model of progressive hypoxia [137,138]. As was mentioned above, the Wayne model does not reproduce the phenomenon of “non-culturability”, and thus it does not represent the resuscitation of “non-culturable” bacilli during the activation of latent TB in humans and animals well. Nevertheless, the re-aeration of dormant cells in the Wayne model of progressive hypoxia has been quite thoroughly studied to date and may provide some clues to help our understanding of the general mechanisms of *M. tuberculosis*’ exit from dormancy.

### 4.1. Modeling of Resuscitation

The re-aeration of dormant cells in the model of progressive hypoxia. The re-aeration of dormant cells in the Wayne model as a model of the reactivation of latent TB is based on the general idea of eliminating the stress factor that caused dormancy. A hypoxia gradient is known to be established inside an *in vivo* granuloma, with the highest level of hypoxia being in its center [139]. Upon reactivation, bacilli begin to move to the sites with higher oxygen availability, avoiding the granuloma [139,140].

Several experimental variants of this approach have been published, including the agitation of a culture during re-aeration to ensure the availability of oxygen [137,141], providing constant access to oxygen via flasks with air-filters [138,142], etc. Although most investigators did not change the growth media when initiating the process of re-aeration, some groups of scientists changed the media before starting re-aeration [137,138]. Several researchers have studied the re-aeration process after 7 days of hypoxia [141,142,143], and in other studies the re-aeration of cells was examined after 20 or 25 days under hypoxia [114,137,138].

Quite expectedly, the level of the expression of Dos-regulon in the reactivation process was found to be very low. Seventy-two hours after the start of re-aeration, almost all genes of Dos-regulon returned to the basic level [138,143], suggesting that in aerobic conditions the role of this regulon is negligible. The decrease in Dos-regulon expression during re-aeration was accompanied by the rapid activation of central metabolic pathways, including the Krebs cycle, glycolysis, ATP synthesis, aerobic respiration, cell wall synthesis, DNA replication, etc. [137,138], which illustrates the deep physiological transformation of *M. tuberculosis* and its readiness for cellular division after finishing hypoxic conditions. Thus, the transcriptional profile of reactivated cells after hypoxia is very similar to that found in actively dividing logarithmic cells [137,138].

The resuscitation of dormant bacteria with a “zero-CFU” phenotype *in vitro*.

The resuscitation of dormant bacteria with a “zero-CFU” phenotype obtained under potassium deficiency is based on re-introducing potassium ions to the medium [110]. Just as in the Wayne model of progressive hypoxia, eliminating a stress factor that caused dormancy (i.e., removing potassium deficiency) triggered a reversion of dormant bacilli to a culturable state. However, in this model, dormant bacilli were “non-culturable” and required 7 days to restore their ability to divide and grow, and the process of resuscitation was characterized by two phases [110]. The first resuscitation phase was characterized by the constant, albeit low, incorporation of radioactive uracil in the resuscitation bacilli, indicating the start of de novo transcription immediately after the removal of the stress factor, which resulted in significant changes in the *M. tuberculosis* transcriptional profile after the first 24 h of resuscitation. This early response included the transcriptional upregulation of genes encoding enzymes of fatty acid synthase system type I (FASI) and type II (FASII), which are responsible for fatty acid/mycolic acid biosynthesis, and regulatory genes, including *whiB6*, which encode a redox-sensing transcription factor. The second resuscitation phase took place 4 days after the resuscitation onset, i.e., before the start of active cell division, and included the activation of central metabolism, e.g., NADH dehydrogenases, ATP-synthases, the Krebs cycle, glycolysis, ribosomal proteins, etc. Remarkably, the activation of the central metabolism coincides with an increase in the intact 23S rRNA and a corresponding decrease in the 23S fragment amount, which was likely a product of a specific 23S rRNA cleavage characteristic for “non-culturable” *M. tuberculosis* [41]. This tendency is most pronounced at day 7, where the integrity of the 23S rRNA is highest and cell multiplication starts. Thus, the resuscitation of dormant “non-culturable” *M. tuberculosis* is characterized by the immediate activation of de novo transcription followed by the upregulation of genes controlling key metabolic pathways and cell multiplication [110].

The reactivation of dormant mycobacteria in a human granuloma *in vitro* model. The formation of a granuloma, which is a multi-cellular immune structure, is known to be a general defense mechanism against *M. tuberculosis* infection in the human host. While it prevents the development of active TB, a granuloma remains a potential reservoir for TB recurrence [144,145]. An *in vitro* granuloma model in which *M. tuberculosis* can subsequently resuscitate under conditions that mimic the weakening of the immune system has been reported recently [146]. The treatment of these *in vitro* granulomas with immunosuppressing anti-tumor necrosis factor-alpha (TNF-α) monoclonal antibodies [146] caused the resuscitation of *M. tuberculosis* in the same way that it does in infected humans [146]. Remarkably, a *lipY* deletion mutant with a compromised ability to mobilize the stored triacylglycerides was unable to resuscitate from dormancy after the treatment of granuloma with anti-TNFα antibodies [146]. Interestingly, a triacylglycerol synthase 1 deletion mutant (*Δtgs1*) with an impaired ability to accumulate triacylglycerides prevented *M. tuberculosis* from transitioning to dormancy [146]. The authors believed that an *in vitro* model of human tuberculosis granuloma mimics the functional features of dormancy and resuscitation observed in human tuberculosis.

The reactivation of dormant *in vitro M. tuberculosis* in mice. A recent study modeled the process of the resuscitation of dormant *M. tuberculosis* in mice. Dormant cells were obtained *in vitro* under acidification with the partial or complete loss of the colony-forming ability [147]. Mice with different genetic susceptibilities to *M. tuberculosis* (highly sensitive I/St mice and relatively resistant B6 mice) were infected with these “non-culturable” mycobacteria. Predictably, dormant cells that experienced difficulties with resuscitation *in vitro* also lacked the ability to divide *in vivo* even in genetically susceptible I/St animals [147].

These researchers continued their efforts to model persistent tuberculosis and its reactivation in animals. Recently, they performed an intriguing study in which they used *M. tuberculosis* mutant strains lacking 4 of the 5 Rpf genes (ΔACDE) to infect mice [148]. It has been demonstrated that *M. tuberculosis* strains lacking three of the five genes belonging to the *rpf* family show seriously attenuated growth *in vivo*, and the quadruple *Rpf* deletion mutant ΔACDE causes defective growth in mouse lungs after aerosol infection [149,150]. Here, the authors compared the reactivation of ΔACDE in mice with polar susceptibility. They found that *M. tuberculosis* ΔACDE progressively proliferated only in I/St lungs. Meanwhile, CFU numbers decreased with time in the lungs of B6 mice. In the late phase of infection, TB foci fused in I/St lungs, resulting in extensive pneumonia, whereas pathology was limited to condensed foci in B6 lungs.

The Cornell model of paucibacillary infection. Undoubtedly, *in vivo* models are a preferable tool for understanding the process of the reactivation of latent infections in detail as they provide the necessary multi-factor aspect of the reactivation process. In the Cornell model, where the antibiotic treatment of infected mice results in CFU decline in organs down to zero, bacteria start to spontaneously reactivate after the end of the treatment [54,151]. The experimental approach implemented in the Cornell model has been expanded to several mouse lines with different susceptibilities to *M. tuberculosis* [27,28,152], as well as to experiments on guinea pigs [29] and rabbits [31,75]. It was found that various immunosuppressive regimens can be used to increase the rate of reactivation of latent infection [59]. However, *in vivo* studies on the phenomenon of the reactivation of *M. tuberculosis* have been more focused on the phenomenon of clinical latency than on the reactivation of infection or the mechanisms underlying resuscitation.

*In vivo *models in primates. The non-human primate model, while it better reflects LTB in humans, is rarely used to study the reactivation process for ethical reasons because the number of animals required to achieve statistically significant experimental results is very high. To stimulate the reactivation of dormant cells in non-human primates, two main strategies are used: (1) TNF-α blocking therapy [33,99], which mimics the effects of some medications used for the treatment of autoimmune diseases in humans that are associated with the risk of tuberculosis reactivation in the case of latent infection; and (2) Co-infection with the simian immunodeficiency virus (SIV) [32,33,136,153], which is used to model HIV-infection in primates.

Co-infection with HIV and *M. tuberculosis* is known to be a serious problem for human health, and increases the risk of the reactivation of *M. tuberculosis* from 10% during the patient’s lifetime to 10% per year [21,22]. This makes the reactivation of *M. tuberculosis* during SIV coinfection especially relevant for the study of LTB progression in humans. When primates are infected with a low dose of *M. tuberculosis* and subsequently develop LTB, the spontaneous activation of the infection has been observed for a small percentage of experimental animals [80,136,154], but the proportion is too low to be practically used as a model of experimental reactivation. The model of *M. tuberculosis*/SIV co-infection increases the percentage of reactivation to approximately 65% and is widely used as an indicator of the successful sterilization of *M. tuberculosis* from the lungs and to study immune reactions in animals; however, the number of studies performed to investigate the process of mycobacterial reactivation is low [32,80,99,136,154,155,156].

### 4.2. Factors Involved in Reactivation

Studies on the process of the reactivation of *M. tuberculosis in vivo* have mainly focused on the phenomenon itself or the observations of the host immune responses of macroorganisms rather than on the mechanisms of bacterial survival in the absence of cell division. A study on the reactivation of hypoxic culture in the Wayne *in vitro* model was performed in more detail and was summarized in a recent review by Veatch and Kaushal [24]; however, the disadvantages of the single-factor approach involving the re-aeration of *M. tuberculosis* cells after hypoxia should be highlighted.

Nevertheless, several factors that are responsible for the successful reactivation of dormant mycobacteria that are not associated with the availability of oxygen have been identified to date.

Proteins of the Rpf family. Resuscitation promoting factors (Rpfs) were the first mycobacterial proteins found to be associated with the reactivation of dormant cells [87,157]. Initially, Rpf protein was identified in a sterile supernatant of the culture of actively growing *M. luteus* cells, and the addition of this supernatant to dormant *M. luteus* cells led to the restoration of their cultivability [158,159,160]. This suggested that active *M. luteus* cells may secrete a component or components to their environment that helps to resuscitate “non-culturable” forms. *M. luteus* Rpf was isolated from a culture supernatant and it was shown that it represents a protein with a molecular mass of about 19 kDa that caused a several-log increase in CFU during the activation of dormant *M. luteus* cells [161]. Interestingly, this protein also had a resuscitating activity for “non-culturable” cells of other species (Shleeva et al., 2004). Homologs of *M. luteus rpf* have been found in many GC-rich bacteria including *M. tuberculosis* (five genes), *M. leprae* (two genes), *M. smegmatis* (four genes), *M. bovis* (BCG) (five genes), *Corynobacterium glutamicum* (two genes), and several *Streptomyces* species [158].

The inactivation of one individual Rpf protein out of the five in *M. tuberculosis* (termed RpfA–E) did not result in any growth defects *in vitro* or during aerosol infection *in vivo* [162], but the inactivation of three of the five genes encoding proteins of the Rpf family caused a decrease in the virulence of *M. tuberculosis* and made the spontaneous reactivation of “non-culturable” cells *in vitro* impossible [149]. The deletion of three of the five *rpf* genes in *M. tuberculosis* in various combinations also resulted in the inability of cells to divide and grow under stress factors both *in vitro* and *in vivo* [163]. *M. tuberculosis* lacking four of the five Rpf genes (ΔACDE) exhibited seriously attenuated growth *in vivo* [150].

It is known that the proteins of the Rpf family are peptidoglycan hydrolases that play a key role in the reactivation of some dormant actinobacteria, including *M. tuberculosis* [157,164,165]. Moreover, Rpfs are required for the reactivation of dormant “non-culturable” bacilli *in vivo* and for pathogenic processes in a murine model of tuberculosis [148,149], but their direct impact on infection in humans is not well understood. The addition of the culture supernatant of *M. luteus* containing the secreted Rpf protein increased the proportion of *M. tuberculosis* cells isolated from clinical sputum samples. Recently, it was found that the stimulation of mycobacteria by Rpfs increased the culturability of mycobacteria by more than 80% in 20 clinical sputum samples taken before the start of TB chemotherapy, and the proportion of such Rpf-dependent bacteria increased significantly during the treatment period compared to a population of «normal» culturable bacteria, which maintained the ability to form colonies on solid growth media [166]. The results of studies on site-directed mutagenesis show that the hydrolytic activity of Rpf is closely connected to the resuscitating activity and growth-stimulating effect of these proteins [164]. RpfB and RpfE were found to interact with the partner protein RipA, which belongs to a group of peptidoglycan endopeptidases [167]. RpfB and RipA may form a complex, and the deletion of the *ripA* gene results in a significant inhibition of mycobacterial growth, as well as an increase in sensitivity to beta-lactam antibiotics [168].

Kana et al. recently found that there are other components of *M. tuberculosis* culture filtrate that are different from Rpf proteins and that are largely responsible for the stimulation of differentially culturable tubercle bacteria in clinical samples [169]. The authors postulated that this stimulatory activity is most likely the result of a combination of factors; however, their nature has yet to be identified [169].

Muropeptides. The participation of muropeptides, which are products of mycobacterial Rpf-mediated cell wall hydrolysis, in the resuscitation of dormant *M. tuberculosis* has recently been discussed [170,171]. Muropeptides are signaling molecules involved in “host–pathogen” interactions [172] that may participate in the germination of *Bacillus subtilis* endospores via interactions with a specific exogenous receptor, the PASTA domain of the membrane serine/threonine protein kinase PrkC [173]. *M. tuberculosis* has a PrkC homolog, serine/threonine kinase PknB, which is essential for the growth of the pathogen *in vitro* as well as for survival within the host [174]. Although the direct involvement of the protein kinase PknB in signal transduction through muropeptides has not yet been confirmed, the signaling action of Rpf proteins mediated by muropeptides is quite probable. Thus, the products of the hydrolysis of mycobacterial peptidoglycan by a mixture of RpfB and a partner protein, RipA, stimulated the resuscitation of the dormant *M. smegmatis* [171].

cAMP and fatty acids. Recently, Shleeva et al. have found that the reactivation of dormant mycobacteria *in vitro* may be stimulated by the addition of free unsaturated fatty acids [175]. The presence of fatty acids enhanced the cAMP level in reactivating *M. smegmatis*. When cAMP or dibutyryl-cAMP were exogenously added to bacilli instead of fatty acids, they caused the resuscitation of *M. smegmatis* and *M. tuberculosis* dormant cells. Interestingly, a *M. smegmatis* lacking MSMEG_4279, which encodes fatty acid-activated adenylyl cyclase could not be resuscitated by fatty acids, but it was resuscitated using cAMP. A specific inhibitor of adenylate cyclase, 8-bromo-cAMP also prevented the fatty acid-dependent reactivation of dormant mycobacteria. *M. smegmatis* and *M. tuberculosis* strains with hyperexpression of adenylate cyclase were unable to transfer into the dormant “non-culturable” state. Interestingly, the *rpfA* gene was found to be activated in *M. smegmatis* at the beginning of the exponential growth phase following the cAMP increase in the lag phase caused by fatty acid-induced cell activation [175].

Trehalose. It has been demonstrated that dormant *M. smegmatis* cells formed by the gradual acidification of a growth medium *in vitro* are characterized by a significant accumulation of free trehalose [176]. Moreover, cell viability depends on the trehalose accumulation level; cells with a high amount of trehalose survive much better than cells with a low amount. A decrease in free trehalose and an increase in the glucose concentration occurred in the early period of resuscitation due to the activation of trehalase, while the trehalase inhibitor validamycin A negatively influenced the resuscitation of dormant cells [176]. The role of trehalose accumulation in the viability of yeast and fungal spores and trehalose breakdown to exit dormancy and for spore germination was intensively studied several decades ago [177,178]. This work revealed common features of the dormant forms of non-sporulating bacteria and true spores.

## 5. Conclusions

**Figure 2:**
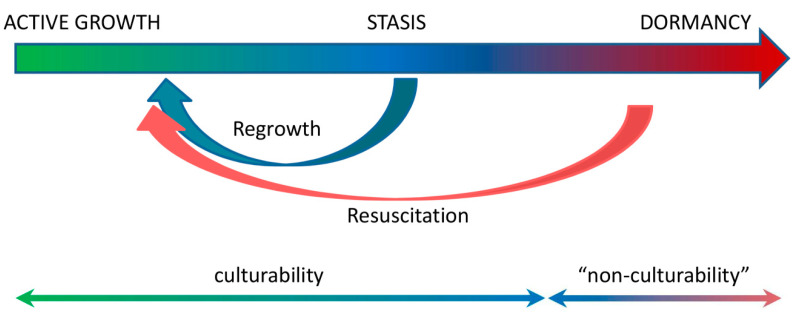
The hypothetical scheme of persisting *M. tuberculosis* population during latent infection.

Notably, a majority of metabolic changes that occur during *M. tuberculosis* dormancy and reactivation identified to date have been described in *in vitro* models, mostly in the Wayne model of progressive hypoxia, rather than in *in vivo* models. This experimental approach can be seriously criticized because the question of the adequacy of such an *in vitro* imitation of mycobacterial persistence in a human host is still open, as the Wayne model does not demonstrate a phenomenon of “non-cultivability”, which is an essential trait of LTB infection in mammalians. In addition, some of the identified biochemical changes, e.g., activation of Dos regulon and glyoxylate shunt and inhibition of ATP synthesis and NADH dehydrogenase, etc., are probably not specific to *M. tuberculosis* persistence. These changes may rather be a part of a general strategy of survival that maintains cell viability under nutrient deficiency and unwelcoming conditions in general. We believe that the persisting *M. tuberculosis* population during latent infection presents a continuum of bacilli that differ in their ability to restore cell division and metabolic activity. Some of them are fully culturable and can divide very slowly under control of the host immune system (stasis), while others are dormant and “non-culturable”, and require a special procedure of resuscitation to revert to active growth (Figure 2). A range of factors that are responsible for the successful resuscitation of dormant “non-culturable” mycobacteria, e.g., proteins of the Rpf family, muropeptides, trehalose, cAMP, and fatty acids, have been identified that may affect new approaches to the therapy and control of LTB infection. The strategy to combat LTB should be directed to the whole spectrum of morphologically and physiologically different *M. tuberculosis* bacilli, which form the persisting population in the human host.

## Figures and Tables

**Table 1:** The spectrum of TB dormancy models *in vitro*.

Model	Isoniazid Tolerance	Rifampicin Tolerance	Changed Morphology	“Non-Culturability”	Need for Resuscitation
Progressive hypoxia [37]	+	-	-	-	-
Nutrient deficiency [86]	+	-	-	-	-
Multiple stress [14]	+	+	-	-	-
ss18b model [88]	+	-	-	+	-
Gradual acidification [89]	ND	+	+	+	+
“Non-culturability” under K^+^-deficiency [40]	+	+	+	+	+
